# Can a Corn-Derived Biosurfactant Improve Colour Traits of Wine? First Insight on Its Application during Winegrape Skin Maceration versus Oenological Tannins

**DOI:** 10.3390/foods9121747

**Published:** 2020-11-26

**Authors:** Giulia Scalzini, Alejandro López-Prieto, Maria A. Paissoni, Vasileios Englezos, Simone Giacosa, Luca Rolle, Vincenzo Gerbi, Susana Río Segade, Benita Pérez Cid, Ana B. Moldes, Jose M. Cruz

**Affiliations:** 1 Dipartimento di Scienze Agrarie, Forestali e Alimentari, Università degli Studi di Torino, Largo Paolo Braccini 2, 10095 Grugliasco (TO), Italy; giulia.scalzini@unito.it (G.S.); mariaalessandra.paissoni@unito.it (M.A.P.); vasileios.englezos@unito.it (V.E.); simone.giacosa@unito.it (S.G.); luca.rolle@unito.it (L.R.); vincenzo.gerbi@unito.it (V.G.); 2 Chemical Engineering Department, School of Industrial Engineering—Centro de Investigación Tecnológico Industrial (MTI), Campus As Lagoas-Marcosende, University of Vigo, 36310 Vigo, Spain; alexlopez@uvigo.es (A.L.-P.); amoldes@uvigo.es (A.B.M.); jmcruz@uvigo.es (J.M.C.); 3 Department of Food and Analytical Chemistry, Faculty of Chemistry, University of Vigo, Lagoas-Marcosende, 36310 Vigo, Spain; benita@uvigo.es

**Keywords:** wine grapes, biosurfactant, exogenous tannins, colour properties, anthocyanin composition, skin maceration, copigmentation, polymerization

## Abstract

In winemaking, oenological tannins are used to preserve wine colour by enhancing the antioxidant activity, taking part in copigmentation, and forming polymeric pigments with anthocyanins. As a novel processing aid, in this study, a biosurfactant extract was evaluated as a solubilizing and stabilizing agent of anthocyanins in red wine. The biosurfactant extract under evaluation was obtained from a fermented residual stream of the corn milling industry named corn steep liquor (CSL). Two red winegrape varieties (*Vitis vinifera* L. cv. Aglianico and Cabernet sauvignon) were studied for anthocyanin content and profile, and colour traits, during simulated skin maceration for 7 days at 25 °C, as well as polymerization and copigmentation at the end of maceration. A model wine solution was used as a control, which was added either with the CSL biosurfactant or with four different oenological tannins (from grape skin, grape seed, quebracho, and acacia). The results showed that CSL biosurfactant addition improved the colour properties of skin extracts by the formation of more stable compounds mainly through copigmentation interactions. These preliminary results highlighted that the effectiveness of CSL biosurfactant is variety-dependent; however, there is no significant protection of individual anthocyanin compounds as observed for delphinidin and petunidin forms using quebracho tannin.

## 1. Introduction

Perceived colour is an important attribute directly influencing the quality of red wine [1]. This feature can determine the product acceptability by consumers as it is related to wine ‘healthy’ and age. Compositionally, red wines are complex because a wide variety of compounds are extracted from grapes during the maceration process, metabolites are released by yeasts during alcoholic fermentation, and different chemical and enzymatic reactions occur [2,3,4]. Particularly, monomeric anthocyanins are located in the berry skin and they are responsible for the colour of red grapes and resulting wines [5]. These phenolic compounds are extracted in the first stages of maceration, even though their diffusion rate depends on the anthocyanin profile. It is well-known that disubstituted anthocyanins diffuse faster than trisubstituted forms [3]. Nevertheless, they can be easily oxidized, and thus, wine colour protection requires the formation of more stable anthocyanin-derived pigments.

The presence of other phenolic compounds can help to stabilize the colour of red wines by their interaction with anthocyanins. In young wines, non-covalent molecular associations through copigmentation can account for up to 30% of the observed colour [6]. Moreover, condensation reactions between anthocyanins and flavanols can occur either directly or mediated by acetaldehyde, leading to the formation of polymeric pigments [2]. These covalently formed adducts, which represent between 35% and 63% of the total wine colour, are resistant to oxidation and sulphur dioxide bleaching [6]. Some studies have highlighted that the content of different phenolic compounds in grape berries and their extractability into the must during maceration are interrelated [7]. At the same time, the concentration and release of both anthocyanins and flavanols are influenced by several factors such as variety, ripeness degree, berry skin mechanical properties, soil conditions, climate, vintage, and viticultural practices [8,9]. In addition, maceration strategies greatly impact the extractability of phenolic compounds during winemaking [9]. Bearing in mind all these aspects, the anthocyanins/tannins ratio has been proposed as an indicator of polymeric pigment formation, wine colour, and overall wine quality [10].

Nowadays, the addition of exogenous tannins during maceration is an oenological practice used for multiple purposes, such as to promote the formation of anthocyanin-derived pigments and therefore preserving anthocyanins and stabilizing wine colour amongst others [11,12]. A wide range of commercial oenological tannins is available, which differ in phenolic composition, botanical origin, and tannic richness [13]. They usually consist of pure or mixed formulations of hydrolysable and condensed tannins. Hydrolysable tannins include gallotannins coming from gallnuts and tara, as well as ellagitannins from chestnut and oak. Condensed tannins, known as proanthocyanidins, are mainly extracted from grape seeds (procyanidins), from grape skins (prodelphinidins and procyanidins), from quebracho (profisetinidins), from mimosa (prorobinetinidins), and acacia (profisetinidins, prorobinetinidins, and prodelphinidins) [14,15,16].

A novel alternative for wine colour preservation has recently been proposed, which is based on the use of surface-active compounds. Particularly, the protection mechanism of a polysorbate-based chemical surfactant (Tween 20) for anthocyanins may be related to the solubilisation of these pigments within the micelles [17]. Nevertheless, the main disadvantage of using chemical surfactants in foods is their low degradability, being not yet admitted as oenological adjuvants by the International Organisation of Vine and Wine (OIV). Instead, biological surfactants, namely biosurfactants, are less toxic as well as more biodegradable and biocompatible than chemical surfactants and emulsifiers [18]. Biosurfactants are produced by microorganisms through biotechnological processes [19] and they are composed of biomolecules. In the food industry, biosurfactants have been used for different purposes, such as fat stabilization, antifoaming, increased solubility in instant drinks and soups, starch complexation, and protective coatings [20,21]. Among biosurfactants, the extract obtained from corn steep liquor (CSL), which is a spontaneously fermented agri-food residue, is cost-competitive and has an important antioxidant activity due to the presence of phenolic compounds [22]. Additionally, its amphiphilic nature, derived from a hydrophobic tail composed of fatty acids [22,23] and a hydrophilic head containing nitrogen similar to lipopeptides [24], makes possible the solubilisation of a great diversity of compounds.

There is evidence that hydrogen bonds and hydrophobic interactions regulate the association between proanthocyanidins and cell wall material [25]. These interactions occur through hydroxyl groups as well as aromatic and glycosidic oxygen atoms contained in proteins and polysaccharides of cell walls [26]. In this regard, the presence of surfactants may also increase the solubility of these hydrophobic complexes and therefore may promote copigmentation and polymerization reactions. Polysorbates are often used in the food industry to solubilize hydrophobic compounds in water-based products [27].

To our knowledge, a biosurfactant has never been tested during grape skin maceration to improve the colour features of red wines. Therefore, the main aim of this study was to evaluate the effectiveness of the biosurfactant extract obtained from CSL to improve the release and stabilization of skin anthocyanins in the first steps of maceration. Furthermore, four different exogenous tannins extracted from grape seeds, grape skins, quebracho, and acacia were also evaluated because they are commonly used for this purpose during winemaking. For two red winegrape varieties (*Vitis vinifera* L. cv. Cabernet sauvignon and Aglianico), the berry skins were subjected to simulated macerations in presence of each exogenous tannin or CSL biosurfactant to reduce the side-reactions due to the complex wine matrix.

## 2. Materials and Methods 

### 2.1. Chemicals and Standards

Solvents of HPLC-gradient grade, Folin–Ciocalteu reagent, bovine serum albumin, and standards of gallic acid, cyanidin chloride, (−)-epicatechin, and (+)-catechin were supplied by Sigma-Aldrich (St. Louis, MO, USA). Malvidin-3-glucoside chloride standard was purchased from Extrasynthese (Genay, France). The solutions were prepared in deionized water produced by a Milli-Q system (Merck Millipore, Darmstadt, Germany).

### 2.2. Grape Samples

In 2018, whole bunches of *Vitis vinifera* L. cv. Aglianico and Cabernet sauvignon red winegrapes were harvested at ripeness (about 24 Brix) from the CNR-IPSP (Consiglio Nazionale delle Ricerche- Istituto per la Protezione Sostenibile delle Piante) ampelographic collection of Grinzane Cavour (Cuneo province, north-west Italy, 44.651 N, 7.995 E). Once in the laboratory, ten kilograms of berries were manually separated from the stalks by cutting the pedicel of each single berry in the proximity of the receptacle. For each grape variety, a set of 200 berries was randomly sampled (“unsorted” samples) for the determination of the grape must standard compositional parameters. For the simulated maceration tests, the remaining berries were density sorted by flotation in different saline solutions (from 130 to 190 g/L NaCl corresponding to densities between 1087 and 1125 kg/m^3^) as described by Fournand et al. [28]. Only the berries belonging to the most representative density class for each variety were selected, corresponding to 1106 kg/m^3^ for Aglianico and 1100 kg/m^3^ for Cabernet sauvignon. Sorted berries were washed with water and visually inspected before analysis, those with damaged skins were discarded. The use of density-sorted berries minimizes the differences in grape berry ripeness within the vineyard. For the chosen density class, two subsamples of 200 berries each were randomly taken to determine the grape must standard compositional parameters and whole grape phenolic ripeness indices. Additionally, three sets of 10 sorted berries were randomly selected to determine total skin phenolic composition, and other eighteen sets of 20 sorted berries were used for skin simulated maceration tests.

### 2.3. Standard Chemical Parameters

The compositional parameters of grape must, which are usually used to define technological ripeness, were determined for each variety in unsorted and sorted samples. Two replicates of about 100 grape berries were manually crushed and the liquid must was centrifuged at 3000× *g* for 15 min at 20 °C, using a Hettich 32R centrifuge (Tuttlingen, Germany). The supernatant obtained was used for analysis. Total soluble solids (Brix) were evaluated using an Atago Palette 0–32 Brix refractometer with automatic temperature compensation (Atago Corporation, Tokyo, Japan). Titratable acidity (expressed as g/L of tartaric acid) and pH determinations were conducted using OIV methods [29] by titrimetry and potentiometry with an InoLab 730 calibrated pHmeter (WTW, Weilheim, Germany), respectively. Reducing sugars (glucose and fructose) were quantified (g/L) using an HPLC (Agilent Technologies, Santa Clara, USA) equipped with a refractive index detector [30].

The two phenolic ripeness indices, cell maturity index (EA%) and seed maturity index (Mp%), were assessed on two replicates of 100 berries, for which grapes were homogenized by grinding according to the method proposed by Saint-Cricq et al. [31] with slight modifications [5]. 

### 2.4. Total Extraction of Phenolic Compounds from Berry Skins

For each variety, three replicates of ten sorted berries were randomly selected. For each replicate, the berries were weighed and the skins were manually separated from the pulp, weighed, and quickly immersed into 25 mL of a buffer solution at pH 3.40 containing 14% v/v of ethanol, 5 g/L of tartaric acid, and 2 g/L of sodium metabisulphite [9]. The extract was obtained by homogenization with an Ultra-Turrax T25 high-speed homogenizer (IKA Labortechnik, Staufen, Germany) for 1 min at 8000 rpm, and subsequent centrifugation at 3000× *g* for 15 min at 20 °C in the Hettich 32R centrifuge. The supernatant obtained was used for the analytical determination of the skin phenolic composition as indicated below. 

### 2.5. Oenological Tannins and Biosurfactant

Four different condensed oenological tannins were considered in this study as representative of the various formulations on the market: (i) two proanthocyanidin preparations extracted from grapes, procyanidins from grape seeds and procyanidins/prodelphinidins from grape skins; (ii) other two from exotic woods, prorobinetinidins from acacia (*Mimosaceae* sp.) and profisetinidins from quebracho (*Schinopsis* spp.). All these oenological tannin formulations were characterized as follows. Total phenolic content was determined using the Folin–Ciocalteu assay in a wine-like solution at pH 3.5 (12% v/v of ethanol and 4 g/L of tartaric acid) containing 1 g/L of tannin [13]. The tannin richness, expressed as g of gallic acid/100 g of commercial formulation, was 62.1 ± 1.8 for grape seed-derived tannins, 56.6 ± 1.9 for grape skins, 51.5 ± 3.2 for acacia, and 55.8 ± 6.9 for quebracho (mean ± standard deviation of three replicates).

As a novel alternative, a biosurfactant was also evaluated for its surface-active and antioxidant properties [22]. The biosurfactant under evaluation comes from a corn steep liquor (CSL), which is a residual stream produced by the corn wet-milling industry, spontaneously fermented by lactic acid bacteria. *Lactobacillus* strains are defined by the US Food and Drug Administration (FDA) as “Generally Recognized As Safe” (GRAS) [32]. The biosurfactant extract was obtained by liquid–liquid extraction with ethyl acetate (CSL solution:ethyl acetate, 1:3 v/v), at room temperature for 60 min, followed by subsequent evaporation of the organic phase. In addition to lipopeptides, different phenolic compounds have been identified in the CSL biosurfactant extract, including protocatechuic acid, vanillic acid, caffeic acid, *p*-coumaric acid, ferulic acid, sinapic acid, epicatechin, and quercetin, which are directly related to the antioxidant activity [22].

### 2.6. Skin Simulated Maceration

For each winegrape variety, three replicates of 20 sorted berries were randomly selected for each of the six skin simulated maceration tests conducted (control, four oenological tannins, and CSL biosurfactant) and then treated following the procedure reported by Paissoni et al. [12]. The berries were weighed, manually peeled, and the resulting skins were carefully separated from the pulp, weighed, and quickly immersed into 100 mL of a buffer solution at pH 3.40 containing 5 g/L of tartaric acid (control), in which an established dose of tannin formulation (grape seeds, grape skins, quebracho, or acacia derived) or CSL biosurfactant was previously added as follows. Each tannin formulation was dissolved in 100 mL of warm (40 °C) buffer solution enriched with 2% v/v of ethanol to help solubilisation. Then, 10 mL of the tannin solution were added to 90 mL of the buffer solution (without ethanol) for each replicate. For this reason, the macerating buffer solution also contains 0.2% v/v of ethanol. The dose of each tannin formulation used in this experiment corresponds to the dosage commonly added during maceration in industrial winemaking, that is 4/5 of the maximum recommended dose (20, 25, 22, and 40 g/hL for grape seeds, grape skins, acacia, and quebracho, respectively). For the CSL biosurfactant, a dose of 100 g/hL was used for the trial, which is higher than the critical micellar concentration (about 200 mg/L) to ensure the formation of micelles [33].

To simulate the wine fermentative maceration process, berry skins were macerated for 7 days at 25 °C with progressive addition of 96% v/v ethanol at 6, 24, 48, 72, and 96 h of maceration. Just before each addition, an equal aliquot of sample was taken to maintain constant the volume of the macerating solution. In particular, the ethanol concentration was 2.50, 4.80, 7.10, 10.6, and 14.0% v/v after addition at 6, 24, 48, 72, and 96 h of maceration, respectively. Once the maceration was completed (168 h), the whole liquid extract was taken for a more complete analytical determination. The effect of adding oenological tannin or biosurfactant was evaluated on the colour traits and anthocyanin extraction yield throughout skin simulated maceration, as well as on the phenolic composition at the end of the process, as indicated below.

### 2.7. Phenolic Composition Determination

The phenolic composition was determined through spectrophotometric methods [34] using a UV-1800 spectrophotometer (Shimazdu Corp., Kyoto, Japan). Total anthocyanin (TA) and non-anthocyanin flavonoid (FNA) concentrations were quantified (mg of malvidin-3-glucoside chloride/kg of grape berries and mg of catechin/kg of grape berries, respectively) by diluting the sample with an ethanol:water:37% hydrochloric acid (70:30:1, v/v) solution and subsequent measurement of absorbance at 536−540 and 280 nm. Total phenolic index (IPT) was evaluated (mg of (−)-epicatechin/kg of grape berries) by measuring absorbance at 280 nm of the sample diluted in water. Total phenolic compounds were also determined (mg of gallic acid/kg of grape berries) through the Folin–Ciocalteu (FC) assay. For the determination of FC in the total skin extracts, since the buffer solution had a very high concentration of sulphur dioxide, the 20-diluted samples were submitted to solid-phase extraction (SPE) on C_18_ Sep-Pak cartridge (Waters Corporation, Milford, MA, USA). Proanthocyanidins (PRO) were quantified (mg of cyanidin chloride/kg of grape) according to the Bate–Smith reaction. Monomeric and oligomeric flavanols were determined (mg of (+)-catechin/kg of grape) as Flavanols Reactive to Vanillin (FRV) [35].

The determination of anthocyanin profile was performed with an Agilent 1260 HPLC-DAD system (Agilent Technologies, Santa Clara, CA, USA), using the chromatographic conditions previously reported by Río Segade et al. [9]. Each skin extract was diluted 1:1 with an HCl solution at pH 0.5, filtered through a 0.45 µm PTFE membrane filter, and then injected (50 μL). A LiChroCART analytical column (25 cm × 0.4 cm i.d.) was used, which was purchased from Merck (Darmstadt, Germany) and packed with LiChrospher 100 RP-18 (5 μm) particles supplied by Alltech (Deerfield, IL, USA). The mobile phase consisted of A = formic acid/water (10:90, v/v) and B = formic acid/methanol/water (10:50:40, v/v), working in gradient mode from 28% of solvent B, increased up to 45% of B in 15 min, to 70% in 20 min, and to 90% in 10 min. Individual anthocyanins were quantified at 520 nm and expressed as a percentage, whereas the sum of all individual forms was expressed as mg of malvidin-3-glucoside chloride/kg of grape berries.

At the end of skin simulated maceration (168 h), the formation of polymeric pigments between anthocyanins and tannins was assessed following the method proposed by Harbertson et al. [36]. The combination of a protein precipitation assay (bovine serum albumin protein, BSA) and the traditional bisulphite bleaching was used to distinguish two classes of polymeric pigments: long polymeric pigments (LPP) and small polymeric pigments (SPP), expressed as a percentage. To evaluate the possible non-covalent molecular associations between anthocyanins and other organic molecules, a copigmentation assay was performed following the Boulton method [37]. Copigmentation and free anthocyanins were estimated as a percentage. 

### 2.8. Colour Characteristics Determination

At each maceration sampling point (6, 24, 48, 72, 96, and 168 h), the visible spectra (380–780 nm) of the undiluted samples were acquired using 1 mm optical path cuvettes. Colour intensity (A_420 nm_ + A_520 nm_ + A_620 nm_ on an optical path of 10 mm) and tonality (A_420 nm_/A_520 nm_) values were obtained according to the method OIV-MA-AS2-07B [29]. CIEL*a*b* parameters, namely lightness (L*), red/green colour coordinate (a*), and yellow/blue colour coordinate (b*), were calculated following the OIV-MA-AS2-11 method [29]. The ΔE* parameter defined as colour difference between control and treated samples was calculated as follows: ∆E* = [(∆L*)^2^ + (∆a*)^2^ + (∆b*)^2^]^1/2^ [29].

### 2.9. Statistical Analysis

Statistical analysis was carried out using R statistic software, version 3.6.2 (R Foundation for Statistical Computing, Vienna, Austria). The normality and homoscedasticity of the data were tested for all parameters by using the Shapiro–Wilk’s and Levene’s tests, respectively. For each studied variable distributed normally and with homogeneity in variance, one-way analysis of variance (ANOVA) using the Tukey HSD post-hoc test was used to evaluate significant differences among treatments at the same maceration time or among different maceration times for the same treatment. When populations presented heterogeneity in variance or were not distributed normally, non-parametric tests were performed (Welch-one-way ANOVA test with Games–Howell post-hoc and Kruskal–Wallis test with Conover post-hoc, respectively). Differences were considered statistically significant at *p*-value < 0.05. Principal component analysis (PCA) using the R package ‘factoextra’ [38] was performed to compare the effect of the different treatments conducted on the two varieties studied while minimizing the contribution of different values of chemical parameters by normalization as *z*-scores before multivariate analysis. 

## 3. Results and Discussion

### 3.1. Grape Characterization

The average values of analytical parameters determined at harvest in unsorted samples for the two red winegrape varieties studied were the following: 24.65 Brix, pH 3.29, and 7.31 g/L as tartaric acid for titratable acidity in Aglianico; and 22.65 Brix, pH 3.44, and 6.08 g/L as tartaric acid for titratable acidity in Cabernet sauvignon. Nevertheless, the experiment was conducted on sorted berries to reduce the heterogeneity in the berry characteristics caused by the different ripening evolution in the vineyard [28,39]. For each variety, the berries belonging to the most representative density class were chosen. Table 1 shows the parameters defining the technological and phenolic ripeness. The metabolites that most influence the grape berry density are sugars and organic acids [40] and therefore Aglianico grapes (1106 kg/m^3^ density) were richer in reducing sugars and acids than Cabernet sauvignon (1100 kg/m^3^ density). Regarding phenolic ripeness, both EA% and Mp% indices were quite similar for the two varieties (43.66 and 39.96 for EA%, 75.67 and 69.85 for Mp% in Aglianico and Cabernet sauvignon, respectively).

Aglianico and Cabernet sauvignon red winegrape varieties were used for this study because of their different phenolic profile. Table 1 reports the phenolic composition and anthocyanin profile of berry skins for the two winegrape varieties at harvest. The richest variety in total skin phenolic compounds, anthocyanins, and flavanols was Cabernet sauvignon. Regarding the anthocyanin profile, Aglianico and Cabernet sauvignon were characterized by a high percentage of trisubstituted anthocyanins (70.48% and 61.72%, respectively, as a sum of delphinidin, petunidin, and malvidin glucosides), with a clear prevalence of malvidin-3-glucoside in both the varieties. However, compared to Aglianico, Cabernet sauvignon had a significantly lower percentage of malvidin-3-glucoside but higher one of delphinidin-3-glucoside. Furthermore, there were significant differences in acylated anthocyanins, Aglianico being richer in cinnamoylated forms whereas Cabernet sauvignon is richer in acetylated derivatives. These results agree with those previously published for these varieties [41].

### 3.2. Colour Parameters Evolution during Skin Maceration

The effect of CSL biosurfactant and the four oenological tannins was assessed on the colour of the macerating solutions during the simulated process. The visible spectra acquired at each sampling point were used to calculate colour intensity as the sum of yellow (A_420 nm_), red (A_520 nm_), and blue (A_620 nm_) colour fractions, as well as tonality (the ratio between yellow and red colour fractions), indicating the contribution of the fractions composing the overall colour. The evolution of colour intensity and tonality during skin simulated maceration is shown in Table 2 for each product tested.

Throughout the maceration process, colour intensity showed a similar trend for all the treatments on Aglianico and Cabernet sauvignon varieties. The colour intensity of macerating solutions increased progressively until reaching a maximum value and then decreased in the latter stages of maceration. This maximum was achieved at 72 h for Aglianico and 48 h for Cabernet sauvignon, although the differences found in the colour intensity values between these two maceration times were not significant. In any case, colour intensity increased between 2.1 and 3.2 units in all the samples tested from 6 to 168 h of maceration. Table 2 also shows the different effects of adding biosurfactant and oenological tannins on colour intensity in the two varieties studied during maceration. For Aglianico winegrapes, the highest values of colour intensity found in the macerating solutions were generally found for quebracho-based tannin formulation, even though the increase observed was not always significant with respect to control. Regarding Cabernet sauvignon, the skin extracts had the most intense colour in the presence of CSL biosurfactant, followed by quebracho tannin, with very few exceptions. At the end of maceration (168 h), the two varieties showed a different influence of treatments on the colour intensity values. No significant differences were observed among the treatments tested for Aglianico, whereas the experiment conducted on Cabernet sauvignon highlighted significantly higher values of colour intensity for CSL biosurfactant treated samples when compared to control as well as to grape skin and seed proanthocyanidin tannins. 

It should be also evidenced that, on average, colour intensity values relative to the extracts obtained from the maceration of Cabernet sauvignon skins were lower than those obtained from Aglianico skins, despite the higher concentration of total anthocyanins. It may be due to differences in the anthocyanin profiles of the two varieties during maceration [42].

Regarding tonality (Table 2), its evolution during maceration followed the same trend for both Aglianico and Cabernet sauvignon varieties, independently on the treatment. During the first 24 h of maceration, a decrease in the tonality value was observed, meaning a higher red colour component (A_520 nm_) with respect to the yellow component (A_420 nm_) and, therefore, the macerating solution shifted to a red hue. This value remained fairly constant until 72 h of maceration and then increased probably due to a loss of red colour component. An advantage of the CSL biosurfactant addition, differently from oenological tannins, is that no significant increase in tonality values was observed at any sampling time if compared with the control maceration. In any case, the differences were not significant among treatments and control for the two varieties from 96 h of skin maceration.

**Figure 1:**
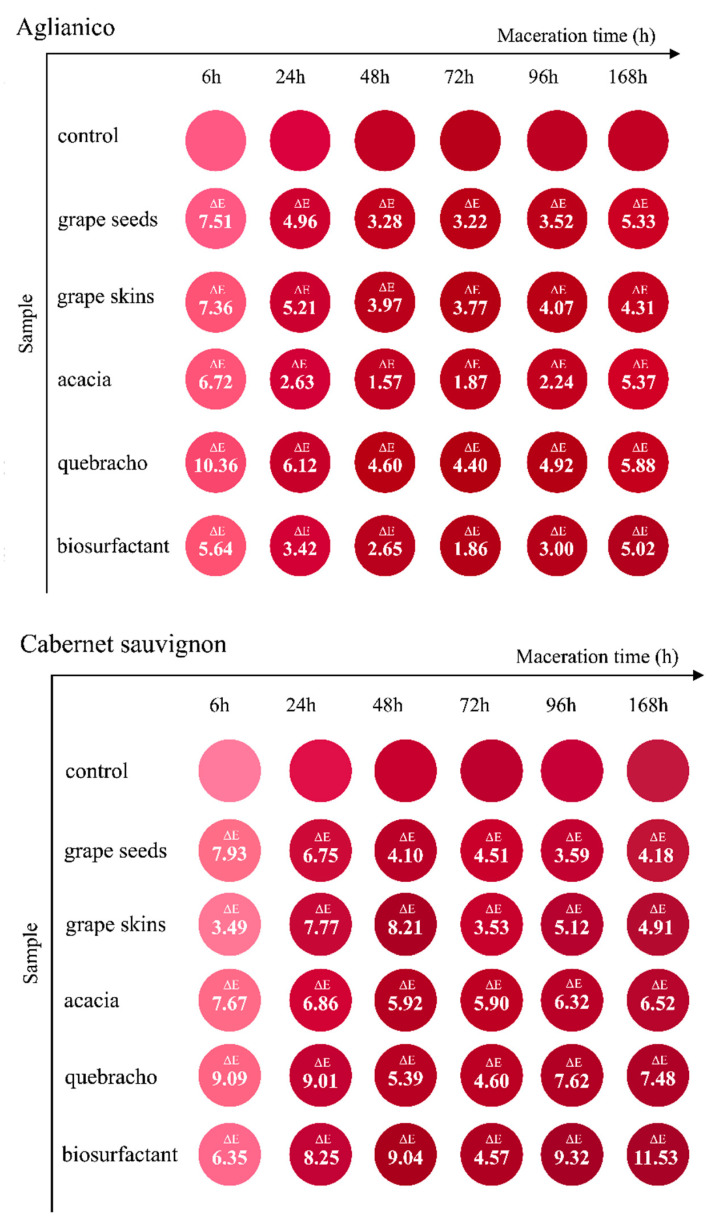
Evolution of the visual colour for different solutions from skin maceration: non-treated control and added with exogenous tannins from different origins (grape seeds, grape skins, acacia, and quebracho) and corn steep liquor (CSL) biosurfactant. Each colour was acquired by spectrophotometry, expressed in CIEL*a*b* coordinates, and converted to RGB (24-bit colour) values. ∆E* values for prefermentative addition *versus* control are shown inside the circle corresponding to visual colour for every sampling point throughout maceration.

To better describe how the addition of the CSL biosurfactant and oenological tannins (grape seeds, grape skins, acacia, and quebracho) affected the visually perceived colour of skin macerating solutions compared to the control during the simulated maceration process, CIEL*a*b* coordinates were calculated (Table S1) and then converted in the corresponding colour on the RGB scale (Figure 1). In agreement with the significant changes observed in colour intensity and tonality (Table 2), CIEL*a*b* coordinates were strongly affected by the skin maceration and treatments tested. Since the RGB space corresponds to the biological processing of colour in the human visual system [43], this representation allows us to visualize the wine colour in a similar way to the real one [44]. For each variety and at each maceration time, objective comparisons were done by quantifying the colour differences found for each treatment in relation to the control using ΔE* values. The results for Aglianico and Cabernet sauvignon varieties are reported in Figure 1. A ΔE* threshold of about three units was established to correctly detect wine colour differences by the human eye [45] or of five units when considering that the colour observation is carried out through a wine taste glass [46].

Figure 1 shows that most of the ΔE* values were greater than 3.0 units, except for acacia tannin at 24, 48, 72, and 96 h of maceration as well as CSL biosurfactant at 48 and 72 h only for Aglianico variety. Nevertheless, some trends can be evidenced. For Aglianico variety, the highest ΔE* values were found at the beginning of maceration (6 h), ranging from 5.64 for CSL biosurfactant to 10.36 for quebracho-based tannin formulation, and, after their decrease until 96 h, the ΔE* parameter increased at the end of maceration up to values between 4.31 for grape skin tannin and 5.88 for quebracho tannin. To understand these variations, the evolution of the three main CIEL*a*b* coordinates was analysed (Table S1). At 6 h of maceration, the differences observed in ΔE* with respect to control corresponded to the increase of b* values (yellow/blue colour coordinate) even though it was significant only for the different tannin formulations tested, evidencing a colour displacement towards yellow hue. Nevertheless, the high ΔE* values observed at 168 h were associated mainly with an increased b* parameter for quebracho tannin (+21%), with a higher value of L* and a* coordinates (lightness and red/green colour coordinate) for acacia tannin (+8 and +5%, respectively), but with a combined decrease of the three CIEL*a*b* coordinates (−11% of lightness, −1% of yellow/blue colour coordinate, and −6% of red/green colour coordinate) for CSL biosurfactant. Therefore, the use of this last product led to the darkest colour macerating solutions (Table S1). Table S2 shows that the colour differences are visible for the use of the CSL biosurfactant not only with respect to the control but also with respect to all the tannin formulations evaluated (ΔE* values from 5.02 to 9.85).

Regarding Cabernet sauvignon, the extracts from the addition of each tannin formulation tested and the CSL biosurfactant showed quantitative differences in the visually perceived colour, compared to the control, as can be observed from ΔE* values above 3.49 for any sampling time (Figure 1). The CSL biosurfactant showed an interesting trend because ΔE* data increased almost progressively during the skin simulated maceration process and reached the highest values at 96 and 168 h of maceration (9.32 and 11.53, respectively).

Particularly, these last two macerating solutions were the darkest despite the reduced red colour component (significantly lower values of L* and a* coordinates, respectively averaged −22% and −11% when compared to control) and had the lowest yellow hue among CSL biosurfactant and tannin added samples (no significant increase of b* colour coordinate with respect to control) (Table S1). The opposite trend was observed for grape seed and acacia tannins, showing a decrease of ΔE* values with the advance of maceration until 48 h and remaining then practically constant (Figure 1). At the end of maceration, the greatest increase in the visually perceived colour (ΔE* values) with respect to control corresponded to CSL biosurfactant, followed by quebracho and acacia tannins. A great increase in ΔE* values (7.66−11.05) for CSL biosurfactant was also observed with respect to all the tannins tested (Table S2). When compared to CSL biosurfactant, both quebracho and acacia tannins led to less dark extracts with more reddish and yellowish hue (Table S1). These results confirmed the differences found in colour intensity (Table 2). 

The impact observed for oenological tannins on colour parameters agrees with previous studies on skin simulated maceration for Aglianico and Cabernet sauvignon winegrape varieties [12]. At 72 h of maceration, colour intensity values for Cabernet sauvignon skins increased with respect to control when quebracho tannin was added. In general, Aglianico tonality seems to be sharply influenced by tannin addition from the beginning of maceration, particularly grape seed tannin formulation led to the greatest increase in tonality values also at 72 h of skin maceration. Other studies have reported that the prefermentative addition of grape seed-derived exogenous tannin has no significant effect on colour intensity and CIEL*a*b* coordinates throughout the winemaking process of red Syrah grapes [47]. Nevertheless, wine colour properties can be diversely influenced by the prefermentative addition of oenological tannins, as found on Sangiovese depending on the initial phenolic concentration of grapes [48].

### 3.3. Anthocyanin Content and Profile during Skin Maceration

**Figure 2:**
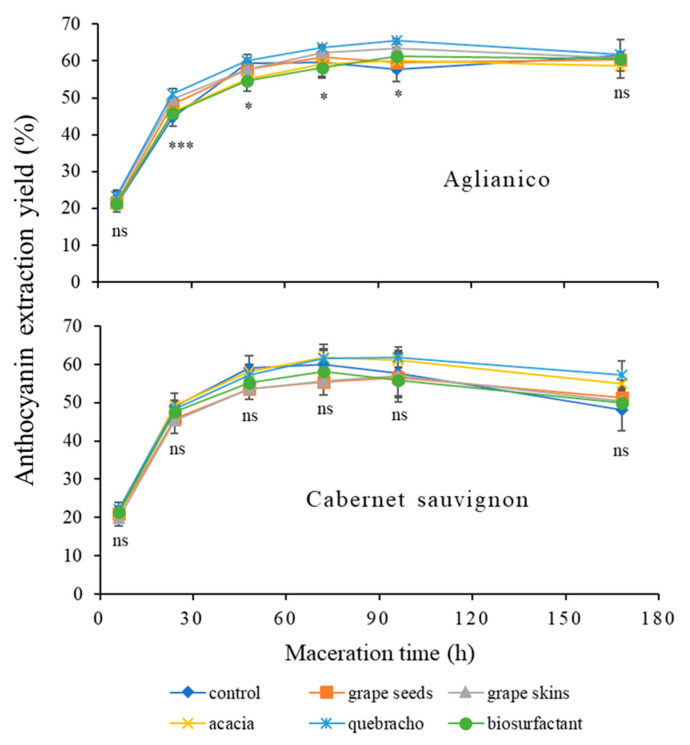
Effect of exogenous tannins and corn steep liquor (CSL) biosurfactant addition on the extraction yield of total anthocyanins during skin simulated maceration. All data are expressed as average value ± standard deviation (*n* = 3). Sign: *, ***, and ns indicate significance at *p* < 0.05, 0.001, and not significant, respectively, for the differences among treatments at each maceration time.

Figure 2 shows total anthocyanin extraction yield for control and tannin added samples throughout the maceration process for Aglianico and Cabernet sauvignon varieties, which was calculated as the ratio between the concentration extracted and that initially present in the berry skins.

Regarding control samples for the two varieties, the evolution of extraction yield was similar until 72 h of maceration. The maximum extraction of total anthocyanins was reached at 72 h and then the extraction yield decreased for Cabernet sauvignon while it was kept practically constant for Aglianico. Although anthocyanins diffuse quickly from the beginning of fermentation as a consequence of their hydrophilic character, trisubstituted forms are released slower into the must than disubstituted forms [3], besides skin structural characteristics influencing the diffusion process [49,50]. For the Aglianico variety, being it richer in cinnamoylated anthocyanins (Table 1), the slower diffusion of these anthocyanin forms may have counterbalanced their possible decrease related to chemical reactions as commented below. The progressive extraction of anthocyanins from berry skins (Figure 2) can explain the higher colour intensity and lower tonality values observed for the extracts sampled between 24 and 72 h of maceration (Table 2). The subsequent decrease of the first parameter and the increase of the second one could be attributable to polymerization reactions rather than to the oxidation of phenolic compounds [12]. 

When the different treatments were compared with respect to control for Aglianico skins, the highest values of total anthocyanin extraction yield corresponded to quebracho tannin, followed by grape skin tannin. Although these differences were significant until 96 h of maceration, then the higher concentration of alcohol tends to minimize them [5]. As reported in Figure 2, once reached the extraction peak, the extraction yield remained practically constant for control and treated samples. For Cabernet sauvignon, it is important to highlight that the addition of quebracho and acacia tannins allowed to reduce slightly the decrease observed in the extraction yield after 72 h of skin maceration, although the differences were not significant. At the end of maceration (168 h), these two treatments increased the anthocyanin extraction yield between +9% and 7% with respect to control.

Table 3 shows the monomeric anthocyanin composition of the skin extracts at the beginning, half, and end of the simulated maceration process (6, 72, and 168 h, respectively) for all the treatments tested and the untreated control on Aglianico and Cabernet sauvignon varieties. Although the different treatments tested did not induce any difference in the total monomeric anthocyanin concentration during skin maceration, with respect to control, quebracho tannin showed concentrations significantly higher than other treatments such as acacia at 72 h of maceration for Aglianico and both the grape-derived formulations at 72 and 168 h for Cabernet sauvignon (Table 3). Moreover, some significant differences were found in the anthocyanin profile of the macerating solutions. The two varieties are malvidin-3-glucoside prevalent, even if the percentage concentrations of the predominant individual forms in the macerating solutions were different from those found in berry skins (Table 1).

Regarding non-acylated anthocyanins for Aglianico variety, the first most abundant form was malvidin-3-glucoside with an average relative concentration of 73.31%, 68.63%, and 67.71% at 6, 72, and 168 h of maceration, respectively. An increase of +14.46%, +10.44%, and +8.89% in the concentration of this compound was found in the control macerating solutions at 6, 72, and 168 h, respectively, when compared to that of grapes. This increase can be attributable to the stability of malvidin-3-glucoside as a consequence of the presence of methoxylated groups in the B-ring [51]. In addition, decreased relative contents of some compounds, such as delphinidin-3-glucoside and petunidin-3-glucoside, occurred, although the addition of quebracho tannin reduced these losses, particularly at the beginning of maceration. In fact, significantly higher delphinidin-3-glucoside and petunidin-3-glucoside contents were found at 6 and 168 h of maceration with respect to the control when quebracho tannin was used (about +0.8% and +0.5%, respectively, for 6 and 168 h). The CSL biosurfactant also preserved delphinidin-3-glucoside and petunidin-3-glucoside but their relative abundances were not significantly different from those of control samples.

The percentages of cyanidin-3-glucoside and peonidin-3-glucoside decreased when maceration progressed at the same time that the malvidin/peonidin ratio increased. Disubstituted anthocyanins are the first diffused from the skins but they are also the most prone to oxidation because of their molecular conformation [3,9]. The same trend was observed for delphinidin-3-glucoside, probably due to its *o*-diphenolic structure, just like cyanidin-3-glucoside [52]. No treatment tested was effective in preserving disubstituted anthocyanin compounds.

Regarding acylated anthocyanin derivatives, these forms are generally worse extracted than the non-acylated anthocyanins as a consequence of the higher retention by cell wall polymeric material [50]. A lower percentage concentration of cinnamoylated forms was observed in macerating solutions than that of grapes, but it increased progressively during maceration. Malvidin-3-glucoside percentage decreased when cinnamoylated derivatives increased in agreement with other previously published studies [53]. Nevertheless, no treatment significantly affected the concentration of these forms regardless of the maceration time. Finally, quebracho tannin and CSL biosurfactant seem to have slowed down the diffusion of acetylated anthocyanin derivatives in the early stage of maceration, but the relative concentration of these forms increased significantly as maceration progressed when these two treatments were carried out.

In the case of Cabernet sauvignon, some variations were also found in the anthocyanin profile of the extracts obtained from skin simulated maceration with respect to that of the grapes, depending on the ease of anthocyanin extraction (Table 1 and Table 3). As already observed for Aglianico, the most abundant anthocyanin compound was malvidin-3-glucoside with an average relative concentration of 53.11%, 49.43%, and 53.48% at 6, 72, and 168 h of maceration, respectively. In the untreated sample, an increase of +6.28%, +7.39%, and +13.66% was observed at 6, 72, and 168 h of maceration, respectively, with respect to grape berries. Nevertheless, the percentage concentration of malvidin-3-glucoside varied differently during maceration depending on the treatment tested. Untreated samples, as well as the samples treated with grape seed tannin and CSL biosurfactant, showed an increasing trend whereas those added with grape skin, acacia, and quebracho tannins evidenced the opposite trend, even though the differences were not always significant. Delphinidin-3-glucoside and petunidin-3-glucoside showed the highest relative concentrations in the samples treated with acacia and quebracho tannins, this increase in the concentration being greater at the beginning of maceration when compared to control (respectively +2.05% and +1.10% for acacia, +1.51% and +1.00% for quebracho). As already observed in Aglianico, the concentration of cyanidin-3-glucoside and peonidin-3-glucoside decreased significantly throughout maceration and a progressive increase in the malvidin/peonidin ratio was observed regardless of treatment. Acacia-derived tannin allowed us to preserve better also disubstituted anthocyanin forms, although the increase observed in the relative concentration was significant with respect to control only for cyanidin-3-glucoside at 72 h of maceration. The changes observed in the anthocyanin profile during Cabernet sauvignon skins maceration are in agreement with those described by Río Segade et al. [54] for simulated macerations in wine-like solutions and by Gil-Muñoz et al. [55] for the wine at the end of alcoholic fermentation.

During maceration, acylated anthocyanins showed different trends in Cabernet sauvignon depending on the treatment tested, as also observed for malvidin-3-glucoside. All treatments slowed down significantly the diffusion of acetylated anthocyanin derivatives in the early stage of maceration. Then, acetylated anthocyanins decreased in control and grape seed formulation, whereas the percentage concentration of these compounds increased significantly for acacia tannin, and it remained statistically unchanged for grape skin and quebracho tannins as well as CSL biosurfactant. As occurred in the Aglianico variety, a lower relative concentration of cinnamoylated forms was observed in macerating solutions compared to that of grapes. Cinnamoylated derivatives increased throughout maceration and, after 168 h of maceration, all treatments showed relative concentrations higher than the control, even if only significantly for grape skin and quebracho tannins. 

The anthocyanin profile of the two varieties studied is different, hence they responded quite differently to the treatments tested. A recently published study has highlighted that grape cultivar features are strictly connected with the tannin addition efficacy in skin simulated maceration conditions [12]. For Cabernet sauvignon, the only significant effect reported was the higher delphinidin-3-glucoside content at 72 h of maceration for grape seed tannin formulation when compared to the control sample. In contrast, no differences were found in non-acylated anthocyanins for Aglianico with the addition of different exogenous tannins (ellagitannins, quebracho, grape seeds, and grape skins). In the present study, similar results were obtained and small variations in the effectiveness of oenological tannins may be due to the different grape ripeness grades influencing the release of anthocyanin forms that have to be protected [39].

The preservation of extracted anthocyanin forms from the first stages of maceration is of great relevance since they influence the colour stability over time through their participation in several chemical reactions [56]. In skin simulated maceration conditions, the CSL biosurfactant played a protective role on delphinidin-3-glucoside and petunidin-3-glucoside in Aglianico winegrape variety whereas on acylated derivatives in Cabernet sauvignon. Nevertheless, its effectiveness resulted to be slightly less than that corresponding to quebracho tannin. The CSL biosurfactant has a high antioxidant activity derived from its phenolic composition, including protocatechuic acid, vanillic acid, caffeic acid, *p*-coumaric acid, ferulic acid, sinapic acid, epicatechin, and quercetin [22]. However, quebracho tannins are characterized by not only high antioxidant capacity but also fast oxygen consumption, even higher than grape-derived proanthocyanidins [13,57]. 

### 3.4. Phenolic Composition at the End of Maceration

Table 4 shows the phenolic composition of the extracts obtained at the end of the maceration process (168 h) for Aglianico and Cabernet sauvignon berry skins using the different treatments above mentioned. For Cabernet sauvignon variety, the results obtained highlight that the addition of CSL biosurfactant significantly increased the percentage concentration of copigmented anthocyanins, with respect to control, in detriment of free forms. In fact, the greatest richness in copigmented anthocyanins corresponded to the CSL biosurfactant. In Aglianico, the copigmentation phenomenon was not reduced significantly when the CSL biosurfactant was used, contrary to what was observed for grape seed tannin. However, no significant difference was found in the relative concentration of polymeric pigments, particularly long polymeric pigments (LPP), for the two varieties studied, even so, it is possible to evidence that the samples added with acacia tannin, followed by CSL biosurfactant, grape seed, and quebracho tannins, showed a slight increase in polymeric pigments for Aglianico (+1.91%, +1.09%, +0.81%, and +0.72%, respectively, compared to control). These values agree with those previously reported for simulated maceration of Aglianico skins [12]. Furthermore, the highest copigmentation and polymerization percentages are not related to total phenolic compounds, to non-anthocyanin flavonoids, or to total anthocyanins, whose highest concentrations were found in the samples added with quebracho tannin for both the varieties (Table 4).

The red colour of young wines is mainly due to the presence of monomeric anthocyanins, but they are unstable and can be degraded by oxidation. Once extracted from berry skins, they can take part in copigmentation and polymerization reactions forming more stable pigments. In the present study, most anthocyanins (59.6–67.6%) were in the monomeric form, as expected at the first stages of winemaking [34]. An anthocyanin fraction of about 19.9–36.6% consisted of polymeric pigments, with a greater contribution of small polymeric pigments (SPP) than large polymeric pigments (LPP). In fact, polymerization reactions are destined to increase at later stages of winemaking. These polymeric pigments are formed as a result of the reactions between anthocyanins and condensed tannins, starting from the beginning of maceration and increasing during wine ageing [58,59]. The addition of exogenous tannins can promote the formation of polymeric pigments through two mechanisms. The antioxidant activity of these products may preserve grape anthocyanins and tannins that can react together, or oenological tannins can combine directly with released anthocyanins stabilizing colour before endogenous tannins are extracted. In the case of oenological tannins, these mechanisms have been widely studied [13,57]. However, the chemical structure and dosage of the oenological tannins used as well as the ratio of tannins to anthocyanins in the wine influence their effectiveness on colour stabilization. Particularly, an imbalance in the anthocyanin/tannin ratio may favour the tannin polymerization and thus increasing the yellow hue [16]. The CSL biosurfactant is a novel alternative and therefore specific studies are needed. Nevertheless, it can be hypothesized a copigment function with a protective role on wine anthocyanins against oxidation as commented below.

The remaining colour fraction, ranging from 18.4% to 26.1%, corresponds to copigmented anthocyanins. Copigmentation has a positive effect on the wine colour because it helps to stabilize the structure of anthocyanins. With regard to the CSL biosurfactant, this is the first time that its effectiveness on colour preservation was studied on a wine-like solution. Therefore, it is interesting to analyse the differences with respect to untreated (control) and tannin-treated samples, since different tannin formulations are used in winemaking for their positive effects on wine colour stability [16]. A very interesting aspect of the CSL biosurfactant is its ability to increase significantly the relative concentration of copigmented anthocyanins in Cabernet sauvignon variety with respect to control (+6.53%, Table 4), surpassing that of all exogenous tannins evaluated (grape skin, grape seed, acacia, and quebracho). This fact explains the significantly higher values of colour intensity and lower L* coordinate for CSL biosurfactant, when compared to control and grape-derived tannins (Table 2 and Table S1). This improvement in colour properties agrees with a bathochromic shift and hyperchromic effect on absorbance at 520 nm associated with copigmentation, involving a blueness hue [37]. The bathochromic effect occurs as a consequence of the affinity of the copigment for the quinoidal forms of anthocyanins. The hyperchromic effect is due to the formation of the flavylium cation–copigment complex [56]. 

The fact that the CSL biosurfactant allowed to encourage copigmentation reactions may be due to its surface-active properties and its phenolic composition consisting of quercetin, epicatechin, sinapic, ferulic, *p*-coumaric, caffeic, protocatechuic, and vanillic acids [22], which are important cofactors [37]. Therefore, the CSL biosurfactant contains phenolic acids and it can justify that the respective macerating solutions had a concentration of total phenolic compounds (IPT) comparable to the solutions added with tannins (Table 4), despite the low content of non-anthocyanin flavonoids (FNA). Considering that the samples treated with quebracho tannin were the richest in IPT and FNA, the nature of the cofactor is of great importance to promoting copigmentation. In fact, this phenomenon depends on the structure of copigments. Particularly, the planar polyphenolic nucleus of flavonols favours π–π stacking with the planar anthocyanin chromophore [56]. A variety effect was also observed since the CSL biosurfactant did not enhance copigmentation reactions for Aglianico skins when compared to the untreated sample. Nevertheless, it is important to evidence that the percentage concentration of copigmented anthocyanins was higher for the CSL biosurfactant than that found for tannins (+4.08% compared to grape seed tannins). In fact, regarding the tannins tested in this experiment, the percentage concentrations of copigmented anthocyanin forms were not significantly different among them or with respect to control, excepting for the low values associated to grape seed tannin in Aglianico (Table 4). This could be due to the richness in coumaroylated anthocyanins in Aglianico skins, which could diminish the effect of added copigments [60,61]. These results are in accordance with the highest values of colour intensity and lowest L* coordinate reported for the CSL biosurfactant also in Aglianico (Table 2 and Table S1). The combined contribution of copigmentation and polymerization reactions could help to better understand the small improvement in the colour properties for Aglianico on simulated skin maceration in the presence of CSL biosurfactant.

Although it is well known that exogenous tannins influence positively colour copigmentation, their effectiveness as copigments depends on the botanical origin, dose, pH level, and ethanol content as reported for a model wine solution containing malvidin-3-glucoside [62]. Within the same type of copigments, a higher tannin dosage resulted in a greater effectiveness on copigmentation because the copigment concentration increased. Moreover, an increase in pH and ethanol strength reduced the tannin effect on red colour. Therefore, copigmentation occurs mainly during the first days of fermentation [63]. Nevertheless, the higher solubility of some compounds in the wine at higher ethanol concentration may have a countering effect enabling a significant contribution of copigmentation also after fermentation [37].

**Figure 3:**
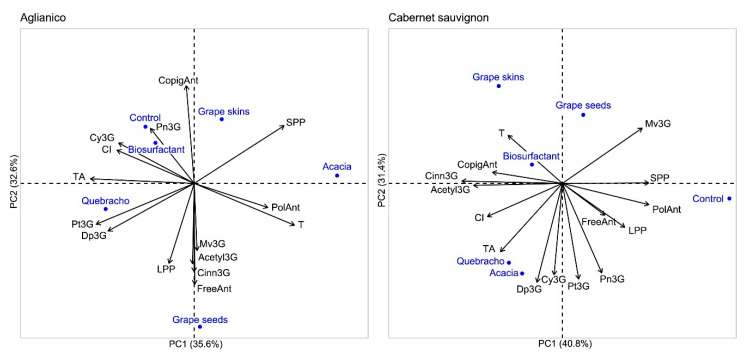
Principal component analysis (PCA) of anthocyanin compounds and colour characteristics of macerating solutions at 168 h of simulated skin maceration for control and for the addition of exogenous tannins and CSL biosurfactant. CI: colour intensity, T: tonality, Dp3G: delphinidin-3-glucoside, Cy3G: cyanidin-3-glucoside, Pt3G: petunidin-3-glucoside, Pn3G: peonidin-3-glucoside, Mv3G: malvidin-3-glucoside, Acetyl3G: acetylated derivatives, Cinn3G: cinnamoylated derivatives, CopigAnt: copigmented anthocyanins, FreeAnt: free anthocyanins, PolAnt: polymerized anthocyanins, LPP: long polymeric pigments, SPP: small polymeric pigments, TA: total anthocyanins.

To better understand the differences among treatments for each variety, a principal component analysis (PCA) was performed (Figure 3). Regarding Aglianico variety, principal component 1 (PC1) accounted for 35.6% of the explained variance, whereas principal component 2 (PC2) explained the 32.6% with a total explained variance by the first two components of 68.2%. PC1 was correlated, in order, with total anthocyanins, tonality, petunidin-3-glucoside (−0.936, 0.903, and −0.883, respectively, all *p* < 0.02), and PC2 was strongly influenced by free and copigmented anthocyanins with a correlation of −0.909 and 0.881 (both *p* < 0.02). Figure 3 confirmed that quebracho tannin protects better anthocyanins and colour with respect to other tannins and CSL biosurfactant, particularly delphinidin-3-glucoside and petunidin-3-glucoside. Moreover, the CSL biosurfactant has not reported differences when compared to control. For Cabernet sauvignon, the multivariate analysis explained 72.2% of the total variance, accounting for PC1 for 40.8% and PC2 for 31.4%. The first principal component was correlated to cinnamoylated and acetylated anthocyanin derivatives (−0.977, *p* < 0.001 and −0.863, *p* < 0.03, respectively), as well as to polymerized pigments and small polymeric pigments (both 0.840, *p* < 0.04). The second principal component was mainly associated with delphinidin-3-glucoside (−0.960, *p* < 0.01), petunidin-3-glucoside (−0.929, *p* < 0.01), cyanidin-3-glucoside (−0.888, *p* < 0.02), and peonidin-3-glucoside (−0.869, *p* < 0.03). As can be observed in Figure 3, all treatments increased total anthocyanin concentration, colour intensity, acylated anthocyanin forms, and copigmented anthocyanins with respect to control. CSL biosurfactant showed an intermediate improvement between that of tannins from exotic oak (acacia and quebracho) and that corresponding to grape-derived tannins (skins and seeds) regarding the preservation of individual anthocyanins, but it was more strongly related to copigmented anthocyanins.

## 4. Conclusions

Anthocyanins are phenolic compounds responsible for the colour of red wine, which is the first attribute perceived by consumers and a major factor determining the quality. These red pigments are released in the first steps of maceration from grape skins and they can undergo chemical reactions influencing colour stability. This study has highlighted that the prefermentative addition of a biosurfactant from a corn steep liquor (CSL), which is a residual stream of the corn wet-milling industry, could represent a promising tool in order to improve colour properties of young red wines. Its effectiveness was variety dependent on skin simulated maceration conditions. After 168 h of maceration, a higher colour intensity was observed in agreement with lower values of lightness (L* colour coordinate). These colour differences can be visualized as shown by the high values of ΔE* parameter, achieving 5.02 and 11.53 units for Aglianico and Cabernet sauvignon, respectively, with respect to untreated samples. The more significant improvement in colour properties for the second winegrape variety seems to be mainly due to the copigmentation effect rather than the protection of specific individual anthocyanin forms, for which quebracho tannin resulted to be more effective regarding delphinidin-3-glucoside and petunidin-3-glucoside. Instead, a combined contribution of copigmentation and polymerization reactions could justify the improved colour of Aglianico skin extracts induced by the CSL biosurfactant. The knowledge of the effectiveness of CSL biosurfactant to preserve the wine colour may open a new field of research on its potential for winemaking. Future research will focus on evaluating the effectiveness of the CSL biosurfactant for colour stability during a real winemaking and wine ageing.

## Figures and Tables

**Table 1:** Composition of sorted grape berries for Aglianico and Cabernet sauvignon winegrapes.

Compound	Unit	Grape Cultivar		Sign
		Aglianico	Cabernet Sauvignon	
*Grape must* ^a^				
Reducing sugars	g/L	262 ± 4	234 ± 5	*
pH	-	3.30 ± 0.01	3.49 ± 0.00	*
Titratable acidity	g/L as tartaric acid	7.09 ± 0.05	5.68 ± 0.03	*
EA%	%	43.66 ± 1.55	39.96 ± 0.69	ns
Mp%	%	75.67 ± 0.01	69.85 ± 0.48	**
*Grape skin phenolic composition* ^b^				
TA	mg malvidin-3-glucoside chloride/kg grapes	879 ± 15	1060 ± 41	**
IPT	mg (-)-epicatechin/kg grapes	3173 ± 180	3731 ± 178	*
FC	mg gallic acid/kg grapes	1871 ± 298	2671 ± 494	ns
PRO	mg cyanidin chloride/kg grapes	2561 ± 272	4270 ± 185	***
FRV	mg (+)-catechin/kg grapes	462 ± 43	642 ± 80	*
FRV/PRO	-	0.18 ± 0.02	0.15 ± 0.01	ns
*Anthocyanin profile* ^b^				
Dp-3-G	%	5.37 ± 0.27	12.58 ± 0.97	***
Cy-3-G	%	0.30 ± 0.05	1.53 ± 0.32	**
Pt-3-G	%	6.53 ± 0.26	5.32 ± 0.04	**
Pn-3-G	%	2.55 ± 0.30	5.44 ± 0.82	**
Mv-3-G	%	58.58 ± 0.99	43.82 ± 1.38	***
∑ Acetyl	%	3.76 ± 0.13	22.21 ± 0.57	***
∑ Cinnamoyl	%	22.92 ± 1.66	9.10 ± 0.11	***

All data are expressed as average value ± standard deviation (^a^ *n* = 2, ^b^ *n* = 3). Sign: *, **, ***, and ns indicate significance at *p* < 0.05, 0.01, 0.001, and not significant, respectively, according to ANOVA test. EA%: cell maturity index, Mp%: seed maturity index, TA: total anthocyanins, IPT: total phenolic index, FC: Folin–Ciocalteu index, PRO: proanthocyanidins, FRV: flavanols reactive to vanillin. Dp-3-G: delphinidin-3-glucoside, Cy-3-G: cyanidin-3-glucoside, Pt-3-G: petunidin-3-glucoside, Pn-3-G: peonidin-3-glucoside, Mv-3-G: malvidin-3-glucoside.

**Table 2:** Colour intensity and tonality of skin extracts during maceration with tannins from different origins and corn steep liquor (CSL) biosurfactant for Aglianico and Cabernet sauvignon winegrapes.

Colour Index	Grape Cultivar	Treatment	6 h	24 h	48 h	72 h	96 h	168 h	Sign ^b^
Colour intensity (A.U.)	Aglianico	control	1.823 ± 0.187 *γ*	4.110 ± 0.288 b, *β*	5.213 ± 0.313 a, *α*	5.540 ± 0.431 *α*	5.157 ± 0.341 a, *α*	4.847 ± 0.407 *αβ*	***
		grape seeds	2.077 ± 0.218 *γ*	4.423 ± 0.134 ab, *β*	5.367 ± 0.177 a, *α*	5.630 ± 0.177 *α*	5.337 ± 0.175 a, *α*	4.790 ± 0.248 *β*	***
		grape skins	2.203 ± 0.280 *δ*	4.587 ± 0.180 ab, *γ*	5.583 ± 0.050 a, *α*	5.890 ± 0.078 *α*	5.540 ± 0.151 a, *αβ*	5.053 ± 0.257 *βγ*	***
		acacia	2.137 ± 0.203 *δ*	4.243 ± 0.103 ab, *γ*	5.227 ± 0.119 a, *α*	5.433 ± 0.119 *α*	5.137 ± 0.125 a, *α*	4.637 ± 0.108 *β*	***
		quebracho	2.407 ± 0.222 *ε*	4.740 ± 0.131 a, *δ*	5.763 ± 0.120 a, *αβ*	6.103 ± 0.055 *α*	5.723 ± 0.049 a, *β*	5.190 ± 0.085 *γ*	***
		biosurfactant	2.150 ± 0.212 *γ*	4.473 ± 0.219 ab, *β*	5.560 ± 0.295 a, *α*	5.803 ± 0.351 *α*	5.548 ± 0.335 a, *α*	5.323 ± 0.494 *αβ*	***
		*Sign* ^a^	ns	*	*	ns	*	ns	
	Cabernet sauvignon	control	1.477 ± 0.160 c, *β*	3.703 ± 0.349 b, *α*	4.620 ± 0.406 *α*	4.633 ± 0.421 *α*	4.120 ± 0.398 b, *α*	3.703 ± 0.223 c, *α*	***
		grape seeds	1.710 ± 0.100 abc, *δ*	4.003 ± 0.196 ab, *βγ*	4.670 ± 0.108 *α*	4.483 ± 0.333 *αβ*	4.233 ± 0.190 ab, *αβγ*	3.857 ± 0.181 c, *γ*	***
		grape skins	1.540 ± 0.139 bc, *γ*	4.100 ± 0.249 ab, *β*	5.003 ± 0.310 *α*	4.510 ± 0.246 *αβ*	4.443 ± 0.280 ab, *αβ*	4.090 ± 0.274 bc, *β*	***
		acacia	1.757 ± 0.045 abc, *δ*	4.173 ± 0.110 ab, *γ*	5.097 ± 0.234 *α*	5.023 ± 0.280 *α*	4.657 ± 0.092 ab, *αβ*	4.257 ± 0.015 abc, *βγ*	***
		quebracho	1.887 ± 0.117 a, *δ*	4.360 ± 0.215 a, *γ*	5.090 ± 0.278 *α*	5.033 ± 0.301 *αβ*	4.873 ± 0.214 ab, *αβγ*	4.450 ± 0.090 ab, *βγ*	***
		biosurfactant	1.807 ± 0.042 ab, *γ*	4.377 ± 0.012 a, *βγ*	5.490 ± 0.676 *α*	5.217 ± 0.621 *αβ*	4.987 ± 0.434 a, *αβ*	4.637 ± 0.360 a, *αβγ*	*
		*Sign* ^a^	**	*	ns	ns	*	**	
Tonality	Aglianico	control	0.454 ± 0.010 c, *α*	0.397 ± 0.009 b, *β*	0.398 ± 0.006 b, *β*	0.409 ± 0.005 b, *β*	0.435 ± 0.008 *α*	0.478 ± 0.020 *α*	***
		grape seeds	0.513 ± 0.005 a, *α*	0.431 ± 0.010 a, *β*	0.418 ± 0.007 a, *β*	0.428 ± 0.009 a, *β*	0.457 ± 0.008, *β*	0.488 ± 0.010 *β*	***
		grape skins	0.487 ± 0.005 b, *α*	0.416 ± 0.005 ab, *γ*	0.416 ± 0.002 ab, *γ*	0.420 ± 0.003 a, *γ*	0.453 ± 0.005 *β*	0.484 ± 0.007 *α*	***
		acacia	0.511 ± 0.007 ab, *α*	0.428 ± 0.002 a, *β*	0.423 ± 0.006 a, *β*	0.427 ± 0.006 a, *β*	0.460 ± 0.006 *α*	0.491 ± 0.005 *α*	***
		quebracho	0.486 ± 0.012 b, *α*	0.416 ± 0.003 ab, *γ*	0.414 ± 0.003 ab, *γ*	0.418 ± 0.000 a, *γ*	0.451 ± 0.002 *β*	0.476 ± 0.003 *α*	***
		biosurfactant	0.440 ± 0.008 c, *β*	0.396 ± 0.008 b, *γ*	0.397 ± 0.008 b, *γ*	0.408 ± 0.004 b, *γ*	0.433 ± 0.004, *β*	0.477 ± 0.003 *α*	***
		*Sign* ^a^	***	***	***	*	ns	ns	
	Cabernet sauvignon	control	0.460 ± 0.017 c, *βγ*	0.413 ± 0.012 c, *γ*	0.427 ± 0.015 b, *γ*	0.463 ± 0.015 *βγ*	0.490 ± 0.020 *β*	0.570 ± 0.026 *α*	***
		grape seeds	0.537 ± 0.012 a, *β*	0.487 ± 0.025 a, *γδ*	0.483 ± 0.006 ab, *δ*	0.473 ± 0.015 *δ*	0.527 ± 0.006 *βγ*	0.607 ± 0.015 *α*	***
		grape skins	0.510 ± 0.017 ab, *βγ*	0.487 ± 0.021 a, *γδ*	0.487 ± 0.006 a, *γδ*	0.463 ± 0.012 *δ*	0.530 ± 0.010 *β*	0.593 ± 0.012 *α*	***
		acacia	0.517 ± 0.006 ab, *β*	0.460 ± 0.010 ab, *γ*	0.463 ± 0.012 ab, *γ*	0.460 ± 0.010 *γ*	0.507 ± 0.006 *β*	0.597 ± 0.006 *α*	***
		quebracho	0.530 ± 0.017 a, *β*	0.470 ± 0.000 ab, *γ*	0.457 ± 0.006 ab, *γ*	0.457 ± 0.006 *γ*	0.507 ± 0.006 *β*	0.587 ± 0.015 *α*	***
		biosurfactant	0.477 ± 0.015 bc, *β*	0.440 ± 0.017 bc, *β*	0.457 ± 0.047 ab, *β*	0.457 ± 0.038 *β*	0.510 ± 0.026 *β*	0.593 ± 0.015 *α*	***
		*Sign* ^a^	***	*	*	ns	ns	ns	

All data are expressed as average value ± standard deviation (*n* = 3). Sign: *, **, ***, and ns indicate significance at *p* < 0.05, 0.01, 0.001, and not significant, respectively, for the differences among treatments for each maceration time (^a^) and among different maceration times for each treatment (^b^) according to ANOVA, Welch’s ANOVA, or Kruskal–Wallis tests. Different Latin letters within the same column indicate significant differences (^a^) and different Greek letters within the same row indicate significant differences (^b^) according to Tukey HSD, Games–Howell, and Conover’s tests (*p* < 0.05) for ANOVA, Welch’s ANOVA, and Kruskal–Wallis tests, respectively.

**Table 3:** Anthocyanin composition during maceration with tannins from different origins and corn steep liquor (CSL) biosurfactant for Aglianico and Cabernet sauvignon winegrapes.

Grape Cultivar	Time (h)	Treatment	Dp-3-G (%)	Cy-3-G (%)	Pt-3-G (%)	Pn-3-G (%)	Mv-3-G (%)	∑ Acetyl (%)	∑ Cinnamoyl (%)	Total (mg/kg grapes)
Aglianico	6	control	3.49 ± 0.47 b	0.38 ± 0.11	4.78 ± 0.45 b	3.63 ± 0.64	73.04 ± 1.72 *α*	4.45 ± 0.18 a, *αβ*	10.23 ± 1.35 *β*	260 ± 30 *β*
		grape seeds	3.90 ± 0.34 ab	0.38 ± 0.02 *α*	5.14 ± 0.28 ab	3.46 ± 0.37 *α*	73.11 ± 0.59 *α*	4.37 ± 0.24 a, *α*	10.10 ± 0.97 *β*	256 ± 26 *β*
		grape skins	3.81 ± 0.14 ab, *αβ*	0.36 ± 0.02 *α*	5.02 ± 0.12 ab, *β*	3.25 ± 0.10 *α*	73.36 ± 0.82 *α*	3.91 ± 0.62 ab	10.84 ± 1.60 *β*	277 ± 23 *β*
		acacia	3.73 ± 0.11 ab, *α*	0.36 ± 0.06 *α*	4.91 ± 0.14 ab, *γ*	3.40 ± 0.43	73.34 ± 1.30 *α*	3.82 ± 0.15 abc, *β*	10.44 ± 0.74 *γ*	261 ± 23 *β*
		quebracho	4.31 ± 0.18 a, *α*	0.39 ± 0.02 *α*	5.52 ± 0.20 a, *β*	3.44 ± 0.15 *α*	73.15 ± 0.25 *α*	3.35 ± 0.02 bc, *γ*	9.84 ± 0.48 *γ*	294 ± 21 *γ*
		biosurfactant	3.92 ± 0.11 ab, *α*	0.40 ± 0.04 *α*	5.20 ± 0.11 ab, *β*	3.71 ± 0.30 *α*	73.85 ± 0.74 *α*	3.03 ± 0.08 c, *γ*	9.89 ± 0.89 *β*	277 ± 23 *β*
		*Sign* ^a^	*	ns	*	ns	ns	***	ns	ns
	72	control	4.14 ± 0.60 a	0.34 ± 0.08	6.01 ± 0.59 ab	2.90 ± 0.55	69.02 ± 1.91 *αβ*	3.52 ± 0.77 c, *β*	14.07 ± 1.94 *αβ*	694 ± 31 ab, *α*
		grape seeds	4.17 ± 0.51 a	0.26 ± 0.03 *β*	6.03 ± 0.44 ab	2.53 ± 0.13 *β*	68.65 ± 0.15 *β*	4.55 ± 0.18 a, *α*	13.80 ± 0.82 *α*	704 ± 26 ab, *α*
		grape skins	4.24 ± 0.26 a, *α*	0.27 ± 0.02 *β*	5.97 ± 0.21 ab, *α*	2.48 ± 0.13 *β*	68.02 ± 0.27 *β*	4.34 ± 0.15 ab	14.68 ± 0.25 *α*	707 ± 7 ab, *α*
		acacia	3.79 ± 0.07 a, *α*	0.26 ± 0.04 *αβ*	5.50 ± 0.12 b, *α*	2.59 ± 0.43	68.49 ± 0.39 *β*	4.33 ± 0.16 ab, *α*	15.05 ± 0.18 *β*	673 ± 13 b, *α*
		quebracho	4.52 ± 0.20 a, *α*	0.31 ± 0.01 *β*	6.35 ± 0.18 a, *α*	2.67 ± 0.10 *β*	68.22 ± 0.22 *β*	3.90 ± 0.04 bc, *β*	14.02 ± 0.45 *β*	745 ± 4 a, *α*
		biosurfactant	4.09 ± 0.15 a, *α*	0.32 ± 0.03 *αβ*	5.93 ± 0.12 ab, *α*	2.84 ± 0.24 *β*	69.35 ± 0.83 *β*	3.45 ± 0.06 c, *β*	14.01 ± 0.63 *α*	664 ± 27 b, *α*
		*Sign* ^a^	*	ns	*	ns	ns	*	ns	**
	168	control	3.16 ± 0.80 b	0.30 ± 0.02	5.61 ± 0.91 b	2.65 ± 0.56	67.47 ± 1.55 *β*	4.88 ± 0.31 a, *α*	15.93 ± 2.08 *α*	644 ± 67 *α*
		grape seeds	3.41 ± 0.73 ab	0.25 ± 0.06 *β*	5.80 ± 0.83 ab	2.28 ± 0.19 *β*	68.73 ± 0.57 *β*	3.23 ± 0.61 b, *β*	16.33 ± 1.76 *α*	692 ± 50 *α*
		grape skins	3.50 ± 0.38 ab, *β*	0.27 ± 0.02 *β*	5.79 ± 0.51 ab, *αβ*	2.28 ± 0.10 *β*	68.28 ± 0.94 *β*	3.37 ± 1.14 ab	16.51 ± 0.71 *α*	665 ± 21 *α*
		acacia	2.96 ± 0.09 b, *β*	0.24 ± 0.03 *β*	5.20 ± 0.07 b, *β*	2.34 ± 0.42	67.15 ± 0.07 *β*	4.53 ± 0.17 ab, *α*	17.58 ± 0.38 *α*	634 ± 5 *α*
		quebracho	3.74 ± 0.08 a, *β*	0.27 ± 0.01 *γ*	6.02 ± 0.09 a, *α*	2.38 ± 0.08 *γ*	66.97 ± 0.20 *γ*	4.14 ± 0.07 ab, *α*	16.49 ± 0.20 *α*	685 ± 8 *β*
		biosurfactant	3.42 ± 0.28 ab, *β*	0.27 ± 0.02 *β*	5.87 ± 0.32 ab, *α*	2.41 ± 0.11 *β*	67.65 ± 1.85 *β*	3.82 ± 0.07 ab, *α*	16.56 ± 1.74 *α*	646 ± 25 *α*
		*Sign* ^a^	**	ns	**	ns	ns	*	ns	ns
		*Sign* ^b^	ns,ns,*,***,**,*	ns,**,**,*,***,**	ns,ns,*,**,**,**	ns,**,***,ns,***,**	*,***,***,***,***,**	*,*,ns,**,***,***	*,**,**,***,***,**	***,***,***,***,***,***
Cabernet sauvignon	6	control	6.86 ± 0.99 b, *β*	1.66 ± 0.71 ab	5.14 ± 0.36 b, *β*	6.06 ± 1.13	50.10 ± 1.79 b, *β*	26.20 ± 1.99 a, *α*	3.98 ± 0.44 *β*	294 ± 27 *γ*
		grape seeds	7.26 ± 0.70 ab	1.38 ± 0.18 b, *α*	5.70 ± 0.34 ab, *β*	6.33 ± 0.84 *α*	54.28 ± 0.60 a, *α*	20.01 ± 2.00 b, *β*	3.40 ± 0.67 *β*	290 ± 20 *γ*
		grape skins	6.85 ± 0.63 b, *αβ*	1.31 ± 0.19 b, *α*	5.56 ± 0.29 ab, *β*	6.35 ± 0.26 *α*	55.45 ± 1.33 a, *α*	20.84 ± 2.22 b	4.48 ± 0.65 *β*	275 ± 27 *γ*
		acacia	8.91 ± 0.57 a, *αβ*	2.40 ± 0.17 a, *α*	6.24 ± 0.32 a, *β*	7.23 ± 0.54 *α*	53.04 ± 0.37 ab, *α*	18.72 ± 0.39 b, *β*	3.46 ± 0.90 *β*	295 ± 8 *γ*
		quebracho	8.37 ± 0.57 ab, *β*	1.64 ± 0.16 ab, *α*	6.14 ± 0.34 a, *β*	6.54 ± 0.06 *α*	53.00 ± 1.07 ab, *α*	21.28 ± 2.75 ab	3.03 ± 0.66 *β*	294 ± 35 *γ*
		biosurfactant	7.70 ± 0.39 ab, *β*	1.87 ± 0.33 ab, *α*	5.74 ± 0.19 ab, *β*	6.84 ± 0.43 *α*	52.80 ± 0.91 ab, *αβ*	20.81 ± 0.49 b	4.24 ± 0.56 *β*	313 ± 21 *γ*
		*Sign* ^a^	*	*	*	ns	**	**	ns	ns
	72	control	9.50 ± 0.21 ab, *α*	1.32 ± 0.04 b	7.38 ± 0.21 ab, *α*	5.13 ± 0.57	51.21 ± 0.53 a, *β*	19.64 ± 0.83 de, *β*	5.85 ± 0.32 b, *α*	846 ± 24 ab, *α*
		grape seeds	8.27 ± 0.65 b	1.02 ± 0.12 b, *αβ*	6.72 ± 0.28 b, *α*	4.73 ± 0.58 *αβ*	49.31 ± 1.11 ab, *β*	23.92 ± 0.57 a, *α*	6.04 ± 0.63 b, *α*	758 ± 11 b, *α*
		grape skins	8.19 ± 0.61 b, *α*	0.98 ± 0.17 b, *αβ*	6.64 ± 0.33 b, *α*	4.76 ± 0.25 *β*	49.38 ± 1.18 ab, *β*	22.97 ± 0.30 ab	7.09 ± 0.23 a, *α*	762 ± 59 b, *α*
		acacia	10.41 ± 0.63 a, *α*	1.82 ± 0.05 a, *β*	7.36 ± 0.30 ab, *α*	5.43 ± 0.38 *β*	47.01 ± 0.51 b, *γ*	21.59 ± 0.49 bc, *α*	6.38 ± 0.31 ab, *α*	851 ± 19 ab, *α*
		quebracho	10.16 ± 0.69 a, *α*	1.30 ± 0.12 b, *αβ*	7.50 ± 0.21 a, *α*	5.14 ± 0.12 *β*	48.67 ± 0.81 ab, *β*	20.88 ± 0.30 cd	6.35 ± 0.35 ab, *α*	865 ± 33 a, *α*
		biosurfactant	9.57 ± 0.69 ab, *α*	1.39 ± 0.28 ab, *αβ*	7.30 ± 0.28 ab, *α*	5.10 ± 0.33 *αβ*	51.02 ± 1.07 a, *β*	19.12 ± 0.73 e	6.51 ± 0.22 ab, *α*	807 ± 34 ab, *α*
		*Sign* ^a^	**	***	*	ns	**	***	*	**
	168	control	7.19 ± 0.02 abc, *αβ*	0.98 ± 0.03 ab	6.84 ± 0.25 *α*	4.77 ± 0.71	57.48 ± 1.12 a, *α*	17.46 ± 1.73 d, *β*	5.30 ± 0.37 b, *α*	668 ± 11 ab, *β*
		grape seeds	6.55 ± 0.92 bc	0.82 ± 0.14 b, *β*	6.43 ± 0.52 *αβ*	4.48 ± 0.60 *β*	57.24 ± 1.73 a, *α*	18.67 ± 0.60 cd, *β*	5.81 ± 0.22 ab, *α*	591 ± 37 b, *β*
		grape skins	6.35 ± 0.76 c, *β*	0.74 ± 0.15 b, *β*	6.00 ± 0.45 *αβ*	4.20 ± 0.25 *β*	52.34 ± 1.45 bc, *αβ*	23.91 ± 0.22 a	6.45 ± 0.24 a, *α*	579.51 ± 55 b, *β*
		acacia	8.51 ± 0.68 ab, *β*	1.36 ± 0.07 a, *γ*	6.91 ± 0.40 *αβ*	4.88 ± 0.40 *β*	49.73 ± 0.41 c, *β*	22.47 ± 0.65 ab, *α*	6.15 ± 0.44 ab, *α*	657 ± 23 ab, *β*
		quebracho	8.77 ± 0.78 a, *αβ*	1.04 ± 0.13 ab, *β*	7.21 ± 0.29 *α*	4.68 ± 0.12 *γ*	50.27 ± 0.89 c, *β*	21.67 ± 0.26 b	6.36 ± 0.42 a, *α*	711 ± 25 a, *β*
		biosurfactant	7.46 ± 0.90 abc, *β*	0.99 ± 0.24 ab, *β*	6.64 ± 0.39 *α*	4.37 ± 0.38 *β*	53.89 ± 1.24 ab, *α*	20.46 ± 0.90 bc	6.19 ± 0.30 ab, *α*	619 ± 32 ab, *β*
		*Sign* ^a^	*	**	ns	ns	***	***	*	**
		*Sign* ^b^	*,ns,*,*,*,*	ns,*,*,***,**,*	**,*,*,*,**,**	ns,*,***,**,***,***	*,***,**,***,***	**,**,ns,***,ns,ns	*,**,***,**,***,***	***,***,**,***,***,***

All data are expressed as average value ± standard deviation (*n* = 3). Sign: *, **, ***, and ns indicate significance at *p* < 0.05, 0.01, 0.001, and not significant, respectively, for the differences among treatments for each maceration time (^a^) and among different maceration times for each treatment (^b^) according to ANOVA, Welch’s ANOVA, or Kruskal–Wallis tests. Different Latin letters within the same column indicate significant differences (^a^) and different Greek letters within the same column indicate significant differences (^b^) according to Tukey HSD, Games–Howell, and Conover’s tests (*p* < 0.05) for ANOVA, Welch’s ANOVA, and Kruskal–Wallis tests, respectively. Dp-3-G: delphinidin-3-glucoside, Cy-3-G: cyanidin-3-glucoside, Pt-3-G: petunidin-3-glucoside, Pn-3-G: peonidin-3-glucoside, Mv-3-G: malvidin-3-glucoside. Total anthocyanins were expressed as malvidin-3-glucoside chloride.

**Table 4:** Phenolic composition of skin extracts at the end of maceration with tannins from different origins and corn steep liquor (CSL) biosurfactant for Aglianico and Cabernet sauvignon winegrapes.

Grape Cultivar	Treatment	Copigmented Anthocyanins (%)	Free Anthocyanins (%)	Polymeric Pigments (%)	LPP (%)	SPP (%)	IPT (mg/kg grapes)	TA (mg/kg grapes)	FNA (mg/kg grapes)
Aglianico	control	26.13 ± 1.14 a	63.55 ± 0.25 b	19.90 ± 0.63	8.89 ± 0.68	11.01 ± 0.14	2014 ± 148 c	542 ± 57	639 ± 30 c
	grape seeds	21.62 ± 0.72 b	67.57 ± 0.31 a	20.71 ± 0.95	9.95 ± 0.97	10.75 ± 0.02	2285 ± 68 bc	532 ± 19	828 ± 21 b
	grape skins	24.50 ± 0.64 ab	64.37 ± 1.16 ab	20.19 ± 0.35	8.10 ± 1.06	12.09 ± 1.17	2320 ± 89 b	535 ± 5	814 ± 22 b
	acacia	25.24 ± 1.40 ab	64.11 ± 1.14 ab	21.81 ± 0.29	9.44 ± 1.207	12.37 ± 1.21	2283 ± 37 bc	498 ± 13	831 ± 25 b
	quebracho	24.34 ± 3.42 ab	65.32 ± 3.54 ab	20.62 ± 0.34	10.14 ± 0.47	10.48 ± 0.39	2641 ± 18 a	544 ± 6	1045 ± 28 a
	biosurfactant	25.70 ± 1.20 a	64.12 ± 1.66 ab	20.99 ± 1.45	9.44 ± 2.11	11.55 ± 0.72	2141 ± 184 bc	533 ± 46	632 ± 70 c
	Sign	*	***	ns	ns	ns	***	ns	***
Cabernet sauvignon	control	18.42 ± 2.15 b	64.88 ± 0.97 a	36.60 ± 10.66	16.07 ± 11.33	20.53 ± 1.55 a	2192 ± 222 c	510 ± 58 b	482 ± 38 d
	grape seeds	19.98 ± 2.30 ab	64.23 ± 2.75 a	29.20 ± 1.31	13.46 ± 1.49	15.74 ± 0.19 b	2733 ± 139 ab	543 ± 13 ab	713 ± 6 c
	grape skins	22.62 ± 3.82 ab	61.96 ± 3.03 ab	28.83 ± 0.67	12.27 ± 0.92	16.56 ± 0.86 b	2671 ± 51 bc	534 ± 28 ab	751 ± 21 c
	acacia	20.14 ± 1.20 ab	64.93 ± 1.32 a	29.62 ± 0.31	14.53 ± 0.52	15.09 ± 0.38 b	2856 ± 166 ab	582 ± 11 ab	860 ± 19 b
	quebracho	23.30 ± 0.65 ab	63.02 ± 0.66 ab	30.53 ± 1.19	13.79 ± 3.62	16.74 ± 2.54 b	3191 ± 208 a	606 ± 41 a	1145 ± 57 a
	biosurfactant	24.95 ± 1.55 a	59.62 ± 1.92 b	32.41 ± 3.37	16.65 ± 3.14	16.65 ± 0.77 b	2659 ± 238 bc	529 ± 16 ab	406 ± 19 d
	Sign	*	***	ns	ns	**	***	*	***

All data are expressed as average value ± standard deviation (*n* = 3). Sign: *, **, ***, and ns indicate significance at *p* < 0.05, 0.01, 0.001, and not significant, respectively, for the differences among treatments according to ANOVA or Welch’s ANOVA tests. Different Latin letters within the same column indicate significant differences according to Tukey HSD and Games–Howell tests (*p* < 0.05) for ANOVA and Welch’s ANOVA, respectively. LPP: long polymeric pigments, SPP: small polymeric pigments, IPT: total phenolic index expressed as (-)-epicatechin, TA: total anthocyanins expressed as malvidin-3-glucoside chloride, FNA: non-anthocyanin flavonoids expressed as (+)-catechin.

## Supplementary Materials

The following are available online at https://www.mdpi.com/2304-8158/9/12/1747/s1, Table S1: CIEL*a*b* parameters of skin extracts during maceration with tannins from different origin and CSL biosurfactant for Aglianico and Cabernet sauvignon winegrapes, Table S2: ΔE* colour parameter of skin extracts at 168 h of maceration without and with addition of tannins from different origin and CSL biosurfactant for Aglianico and Cabernet sauvignon winegrapes.
