# Supplementary material for: Can a Corn-Derived Biosurfactant Improve Colour Traits of Wine? First Insight on Its Application during Winegrape Skin Maceration versus Oenological Tannins
Source: Foods. 2020 Nov 26;9(12):1747. doi: 10.3390/foods9121747 (PMC7760736; doi:10.3390/foods9121747)
Supplement: Supplementary file 1 [file foods-09-01747-s001.pdf]

Table 1. CIEL*a*b* parameters of skin extracts during maceration with tannins from different origin and CSL biosurfactant for Aglianico and Cabernet sauvignon winegrapes.

Colour index	Grape cultivar	Treatment	6 h	24 h	48 h	72 h	96 h	168 h	$Sign^b$
L*	Aglianico	control	59.96 ± 2.84 α	39.28 ± 1.74 a, β	33.39 ± 0.94 a, γ	31.35 ± 1.92 ab, γ	32.92 ± 1.77 a, γ	33.39 ± 1.39 ab, γ	***
		grape seeds	57.01 ± 2.92 α	36.91 ± 1.33 ab, β	33.06 ± 0.72 ab, $\beta\gamma$	31.53 ± 0.87 ab, β	32.41 ± 0.44 a, $\beta\gamma$	35.28 ± 1.18 ab, $\beta\gamma$	***
		grape skins	55.00 ± 3.94 α	36.05 ± 1.21 ab, β	31.92 ± 0.08 ab, $\beta\gamma$	30.33 ± 0.63 ab, γ	31.21 ± 1.24 a, γ	33.41 ± 2.50 ab, $\beta\gamma$	**
		acacia	55.56 ± 2.57 α	37.58 ± 0.33 ab, β	33.12 ± 0.66 ab, $\gamma\delta$	32.04 ± 0.55 a, δ	33.14 ± 0.78 a, $\gamma\delta$	36.05 ± 0.64 a, $\beta\gamma$	***
		quebracho	52.07 ± 2.57 α	34.98 ± 0.84 b, $\beta\gamma$	30.87 ± 0.69 b, δ	29.27 ± 0.33 b, ε	30.38 ± 0.31 a δ	33.31 ± 0.73 b, β	**
		biosurfactant	55.48 ± 2.79 α	37.22 ± 1.60 ab, β	31.61 ± 1.43 ab, $\beta\gamma$	30.20 ± 1.85 ab, γ	30.77 ± 1.73 a, γ	29.80 ± 3.01 ab, γ	***
		$Sign^a$	ns	*	*	*	*	*	
	Cabernet sauvignon	control	65.26 ± 2.61 a, α	41.54 ± 2.02 a, β	35.17 ± 1.90 γ	33.64 ± 2.08 γ	36.04 ± 2.18 a, $\beta\gamma$	37.00 ± 1.13 a, $\beta\gamma$	***
		grape seeds	61.94 ± 1.65 abc, α	37.81 ± 2.39 ab, β	33.07 ± 1.11 β	35.50 ± 3.13 β	34.97 ± 1.58 ab, β	35.96 ± 1.47 ab, β	**
		grape skins	64.35 ± 2.13 ab, α	36.46 ± 1.76 b, β	29.97 ± 1.60 γ	35.21 ± 0.95 β	32.64 ± 1.41 abc, $\beta\gamma$	33.66 ± 2.30 ab, $\beta\gamma$	***
		acacia	60.86 ± 0.85 abc, α	37.80 ± 0.92 ab, β	31.46 ± 1.49 γ	32.85 ± 2.15 γ	32.80 ± 0.59 abc, γ	33.16 ± 0.61 ab, γ	***
		quebracho	58.67 ± 1.66 c, α	34.99 ± 1.29 b, β	31.58 ± 1.77 $\beta\gamma$	32.21 ± 1.71 $\beta\gamma$	30.64 ± 0.83 bc, γ	31.43 ± 0.44 bc, $\beta\gamma$	***
		biosurfactant	59.84 ± 0.70 bc, α	35.26 ± 0.98 b, β	28.84 ± 4.94 β	30.26 ± 4.86 β	28.97 ± 3.25 c, β	27.94 ± 2.94 c, β	***
		$Sign^a$	**	**	ns	ns	**	***	
a*	Aglianico	control	54.14 ± 2.51 γ	63.86 ± 0.95 α	61.62 ± 0.48 $\alpha\beta$	60.19 ± 1.33 β	61.19 ± 0.46 $\alpha\beta$	61.01 ± 0.26 ab, $\alpha\beta$	***
		grape seeds	55.00 ± 1.16 γ	62.00 ± 1.34 $\alpha\beta$	61.53 ± 0.61 $\alpha\beta$	60.59 ± 0.96 β	61.00 ± 0.49 β	63.66 ± 0.76 ab, α	***
		grape skins	56.46 ± 1.45 β	62.12 ± 0.73 α	60.74 ± 0.08 $\alpha\beta$	59.90 ± 0.75 $\alpha\beta$	60.04 ± 1.30 $\alpha\beta$	62.16 ± 2.62 ab, α β	*
		acacia	55.70 ± 1.34 γ	62.58 ± 0.10 $\alpha\beta$	61.40 ± 0.58 β	61.03 ± 0.47 β	61.42 ± 0.64 β	64.11 ± 0.41 a, α	***
		quebracho	58.40 ± 1.58 γ	62.11 ± 0.64 $\alpha\beta$	60.33 ± 0.64 $\beta\gamma$	59.38 ± 0.33 γ	59.94 ± 0.30 $\beta\gamma$	63.16 ± 0.76 ab, α	***
		biosurfactant	57.31 ± 1.22 β	63.00 ± 1.50 α	60.18 ± 1.37 $\alpha\beta$	59.17 ± 0.10 $\alpha\beta$	59.38 ± 1.59 $\alpha\beta$	57.52 ± 2.77 b, β	*
		$Sign^a$	ns	ns	ns	ns	ns	**	
	Cabernet sauvignon	control	48.58 ± 3.22 γ	63.25 ± 0.87 a, α	60.99 ± 1.14 a, α	58.57 ± 1.10 $\alpha\beta$	58.86 ± 1.39 a, $\alpha\beta$	55.25 ± 2.50 a, β	***
		grape seeds	49.89 ± 1.52 β	59.06 ± 2.47 ab, α	57.58 ± 0.95 ab, α	61.03 ± 2.83 α	57.87 ± 0.76 a, α	54.69 ± 0.08 a, β	**
		grape skins	48.88 ± 2.73 β	57.71 ± 2.12 b, $\alpha\beta$	54.64 ± 0.75 b, β	60.90 ± 0.19 α	55.54 ± 0.49 ab, β	53.24 ± 1.24 a, β	***
		acacia	51.15 ± 0.11 γ	60.99 ± 1.51 ab, α	57.54 ± 1.73 ab, $\alpha\beta$	59.89 ± 2.48 α	57.53 ± 1.17 a, $\alpha\beta$	54.23 ± 1.46 a, $\beta\gamma$	***
		quebracho	51.61 ± 2.17 γ	58.11 ± 0.37 b, α	58.06 ± 1.76 ab, α	59.12 ± 1.36 α	55.59 ± 0.19 ab, $\alpha\beta$	53.30 ± 1.29 a, $\beta\gamma$	***
		biosurfactant	51.22 ± 0.60	58.33 ± 1.98 b	54.87 ± 5.42a b	56.32 ± 5.34	53.04 ± 3.29 b	48.13 ± 2.52 b	ns
		$Sign^a$	ns	*	*	ns	**	**	
b*	Aglianico	control	6.07 ± 0.80 b, γ	21.00 ± 2.14 β	27.39 ± 2.41 ab, α	28.54 ± 1.84 b, α	27.30 ± 2.22 ab, α	26.37 ± 2.51 bc, $\alpha\beta$	***
		grape seeds	12.93 ± 1.27 a, γ	24.94 ± 0.10 β	30.66 ± 0.97 ab, α	31.73 ± 1.16 a, α	30.78 ± 1.26 ab, α	30.59 ± 1.48 ab, α	***
		grape skins	10.99 ± 1.26 a, γ	24.69 ± 0.70 β	30.98 ± 0.40 a, α	32.15 ± 0.63 a, α	30.81 ± 0.63 ab, α	30.53 ± 1.39 ab, α	***
		acacia	10.91 ± 1.05 a, γ	22.55 ± 1.17 β	28.92 ± 0.55 b, α	30.07 ± 0.56 ab, α	29.52 ± 0.43 b, α	29.87 ± 0.62 abc, α	***
		quebracho	11.27 ± 1.03 a, γ	24.99 ± 0.50 β	31.02 ± 0.28 a, α	32.33 ± 0.10 a, α	31.32 ± 0.34 a, α	31.84 ± 0.11 a, α	***
		biosurfactant	7.43 ± 1.28 b, γ	23.59 ± 0.97 β	28.74 ± 1.33 ab, α	29.59 ± 1.49 ab, α	28.35 ± 1.51 ab, α	26.05 ± 2.06 c, $\alpha\beta$	**
		$Sign^a$	***	ns	*	**	*	**	
	Cabernet sauvignon	control	3.46 ± 0.77 d, β	18.11 ± 2.74 b, α	22.88 ± 2.96 α	21.55 ± 2.70 b, α	18.37 ± 2.82 b, α	16.38 ± 2.38 b, α	***
		grape seeds	10.54 ± 0.37 a, γ	21.86 ± 0.98 ab, β	23.80 ± 0.58 α	24.83 ± 0.64 ab, α	21.65 ± 0.41 ab, β	20.38 ± 0.41 ab, β	***
		grape skins	6.82 ± 0.24 c, γ	20.10 ± 2.01 ab, $\alpha\beta$	22.48 ± 1.17 $\alpha\beta$	23.67 ± 1.62 ab, α	20.27 ± 1.55 ab, $\alpha\beta$	19.36 ± 0.27 ab, β	***
		acacia	9.20 ± 0.42 ab, δ	23.40 ± 0.89 a, $\beta\gamma$	25.95 ± 1.12 $\alpha\beta$	27.24 ± 1.00 a, α	23.64 ± 1.06 a, $\beta\gamma$	21.54 ± 1.37 a, γ	***
		quebracho	8.93 ± 0.71 b, δ	21.54 ± 1.41 ab, γ	25.64 ± 0.94 $\alpha\beta$	25.88 ± 1.20 a, α	22.63 ± 1.39 ab, $\beta\gamma$	20.97 ± 1.23 ab, γ	***
		biosurfactant	5.47 ± 0.34 c, ε	20.20 ± 0.88 ab, $\beta\gamma$	24.96 ± 1.59 α	23.64 ± 1.47 ab, $\alpha\beta$	20.15 ± 1.69 ab, γ	16.52 ± 1.07 b, δ	***
		$Sign^a$	***	*	ns	*	*	*	

All data are expressed as average value ± standard deviation (n=3). Sign: *, **, ***, and ns indicate significance at $p < 0.05$, 0.01, 0.001, and not significant, respectively, for the differences among treatments for each maceration time ([a]) and among different maceration times for each treatment ([b]) according to ANOVA, Welch's ANOVA, or Kruskal-Wallis tests. Different Latin letters within the same column indicate significant differences ([a]) and different Greek letters within the same row indicate significant differences ([b]) according to Tukey HSD, Games-Howell, and Conover's tests ($p < 0.05$) for ANOVA, Welch's ANOVA, and Kruskal-Wallis tests, respectively. L*: lightness, a*: red/green colour coordinate, b*: yellow/blue colour coordinate.

Table S2. ΔE* colour parameter of skin extracts at 168 h of maceration without and with addition of tannins from different origin and CSL biosurfactant for Aglianico and Cabernet sauvignon winegrapes.

Grape cultivar		control	grape seeds	grape skins	acacia	quebracho	biosurfactant
Aglianico	control	0					
	grape seeds	5.33	0				
	grape skins	4.31	2.39	0			
	acacia	5.37	1.15	3.34	0		
	quebracho	5.88	2.39	1.65	3.51	0	
	biosurfactant	5.02	9.40	7.40	9.85	8.82	0
Cabernet sauvignon	control	0					
	grape seeds	4.18	0				
	grape skins	4.91	2.90	0			
	acacia	6.52	3.07	2.45	0		
	quebracho	7.48	4.77	2.75	2.04	0	
	biosurfactant	11.53	11.05	8.18	9.46	7.66	0
